# Privacy-Preserving Restricted Boltzmann Machine

**DOI:** 10.1155/2014/138498

**Published:** 2014-06-24

**Authors:** Yu Li, Yuan Zhang, Yue Ji

**Affiliations:** ^1^Computer Science and Engineering Department, State University of New York at Buffalo, Buffalo, NY 14260, USA; ^2^State Key Laboratory for Novel Software Technology, Nanjing University, Nanjing 210046, China; ^3^Computer Science and Technology Department, Nanjing University, Nanjing 210046, China; ^4^Tian Jia Bing Hall, Nanjing Normal University, Nanjing 210097, China

## Abstract

With the arrival of the big data era, it is predicted that distributed data mining will lead to an information technology revolution. To motivate different institutes to collaborate with each other, the crucial issue is to eliminate their concerns regarding data privacy. In this paper, we propose a privacy-preserving method for training a restricted boltzmann machine (RBM). The RBM can be got without revealing their private data to each other when using our privacy-preserving method. We provide a correctness and efficiency analysis of our algorithms. The comparative experiment shows that the accuracy is very close to the original RBM model.

## 1. Introduction

With the rapid development of information technology and modern network, huge amounts of personal data are generated every day, and people care deeply about maintaining their privacy. Therefore, there is a need to focus on developing privacy-preserving data mining algorithms. With the rapid growth of social networks like Facebook and LinkedIn, increasingly more research will be based on personal data, such as advertising suggestion. In another scenario, doctors always collect patients' personal information before the diagnosis of a disease or the treatment of an illness. However, in order to prevent the leakage of these privacy data, the Health Insurance Portability and Accountability Act (HIPPA) has set up a series of regulations that protect the privacy of individually identifiable health information.

Data mining is an important interdisciplinary field of computer science and has been widely extended to the fields of bioinformatics, medicine, and social networks. For example, when a research institute wants to study the DNA sequence and related genetic diseases, they need to collect patients' DNA data and apply data mining or machine learning algorithms to obtain a relevant model. However, if scientists from other institutes also want to use these DNA sequences, ensuring that the patients' personal information is protected is an example of the problem at hand. In another scenario, some researchers want to combine the personal data from Facebook and LinkedIn to undertake a study. However, neither company wants to reveal the personal information of their subscribers, and they especially do not want to give it to a competitor. Therefore, we propose a privacy-preserving machine learning method to ensure that individuals' privacy is protected.

The restricted Boltzmann machine (RBM) [1] is increasingly being used in supervised or unsupervised learning scenarios, such as classification. It is a variant of the Boltzmann machines (BMs) which is a type of stochastic recurrent neural network invented by Hinton and Sejnowski. It has been used as windows of mel-cepstral coefficients that represent speech [2], bags of words that represent documents [3], and user ratings of movies [4].

In this paper we propose a privacy-preserving method for training the RBM, which can be used for information sharing without revealing personal data from different institutions to each other. We provide a correctness and efficiency analysis of our algorithms. The comparative experiment shows that the accuracy is very close to original RBM model.

The rest of this paper is organized as follows.  Section 2 describes the related work. We introduce the restricted Boltzmann machine, Gibbs sampling, contrastive divergence, and cryptograph scheme in more detail in Section 3. In Section 4, we describe our privacy-preserving method for training the RBM. The analysis of our model is described in Section 5.  Section 6 gives the design of our experiments in detail. Last, Section 7 is the conclusion of this paper.

## 2. Related Work

In [5], Hinton gives a practical guide for training the restricted Boltzmann machine. It is widely used in collaborative filtering [4]. In [6], Agrawal and Srikant and [7] Lindell and Pinkes propose separately that much of future research in data mining will be focused on the development of privacy-preserving techniques. With the development of privacy-preserving data mining techniques, it can be divided into two classes: the randomization-based method like [7] and the cryptograph-based method like [6].

Randomization-based privacy-preserving data mining, which perturbs data or reconstructs the distribution of the original data, can only provide a limited degree of privacy and accuracy but is more efficient when the database is very large. In [8], Du and Zhan present a method to build decision tree classifiers from the disguised data. They have conducted experiments to compare the accuracy of their decision tree with the one built from the original undisguised data. In [9], Huang et al. study how correlations affect the privacy of a dataset disguised via the random perturbation scheme and propose two data reconstruction methods that are based on data correlations. In [10], Aggarwal and Yu develop a new flexible approach for privacy-preserving data mining, which does not require new problem-specific algorithms since it maps the original dataset into a new anonymous dataset.

Cryptograph-based privacy-preserving data mining, which can provide a better guarantee of privacy when different institutes want to cooperate to meet a common research goal, is always subject to its efficiency when the dataset is very large. In [11], Wright and Yang propose a cryptographic-based privacy-preserving protocol for learning the Bayesian network structure. Chen and Zhong [12] present a cryptographic-based privacy-preserving algorithm for backpropagation neural network learning. In [13], Laur et al. propose cryptographically secure protocols for kernel perceptron and kernelized support vector machines. In [14], Vaidya et al. propose a privacy-preserving naive Bayes classifier on both vertically and horizontally partitioned data.

To the best of our knowledge, we are the first to provide a privacy-preserving RBM training algorithm for vertical partitions.

## 3. Technical Preliminaries

In the section, we give a brief review of RBM and the cryptograph method we have used in our privacy-preserving algorithm. First, we introduce RBM and the learning method for the binary unit. Much of the description about RBM and its training method in this section is adapted from [5, 15]. Second, we introduce the cryptograph technology [12] that we have used in our work.

### 3.1. RBM

**Figure 1:**
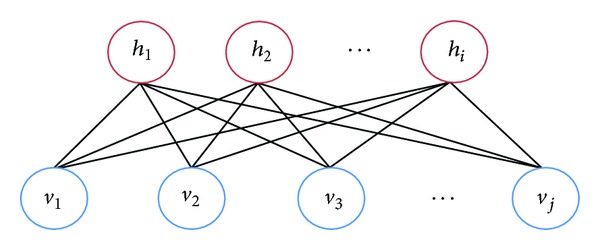
Restricted Boltzmann machine.

The Boltzmann machine (BM) [16] is a stochastic neural network with symmetric connections between units and no connection in the same unit. The BMs can be used to learn important aspects of an unknown probability distribution based on its samples. Restricted Boltzmann machines (RBMs) further restrict that BMs do not have visible-visible and hidden-hidden connections [15], thus simplifying their learning process. A graphical depiction of an RBM is shown in Figure 1. *v*_1_,…, *v*_*j*_ are visible units and *h*_1_,…, *h*_*i*_ are hidden units. All visible units are connected with all hidden units with a weight matrix *W* = {*w*_*ij*_}.

Given *W*, a joint configuration (*v*, *h*) of the visible and hidden units has an* energy* [17] defined as

(1) $$\begin{array}{c} E\left(v,h\right)=-\underset{i\in \text{hidden}}{\sum }c_{i}h_{i}-\underset{j\in \text{visible}}{\sum }b_{j}v_{j}-\underset{i,j}{\sum }h_{i}w_{ij}v_{j}, \end{array}$$

where *v* and *h* are the vectors consisting of states of all visible units and hidden units, respectively; *c*_*i*_ and *b*_*j*_ are the biases associated with unit *i* and unit *j*, respectively, and *w*_*ij*_ is the weight between units *i* and *j*. The energy determines the probability distributions over the hidden units' and visible units' state vectors using an* energy function* as follows:

(2) $$\begin{array}{c} P\left(v,h\right)=\frac{e^{-E(v,h)}}{Z}, \end{array}$$

where *Z* is the sum of *P*(*v*, *h*) for all possible (*v*, *h*) pairs.

### 3.2. RBM with Binary Units

When units' states are binary, according to [18], a probabilistic version of the usual neuron activation function that is commonly studied can be simplified to

(3) $$\begin{array}{c} P\left(h_{i}=1\;|\;v\right)=\text{sigm}\left(c_{i}+W_{i}\cdot v\right),\\ P\left(v_{j}=1\;|\;h\right)=\text{sigm}\left(b_{j}+W_{j}^{\prime }\cdot h\right), \end{array}$$

where sigm denotes the sigmoid function and *W*_*i*_ (and *W*_*j*_′, resp.) is the *i*th row vector (the *j*th column vector, resp.) of *W*.

Based on (2) and (3), the log-likelihood gradients for an RBM with binary units [15] can be computed as

(4) $$\begin{array}{c} -\frac{\partial \log P\left(v\right)}{\partial W_{ij}}=E_{v}\left[P\left(h_{i}\;|\;v\right)\cdot v_{j}\right]-v_{j}^{\left(i\right)}\cdot \text{sigm}\left(W_{i}\cdot v^{\left(i\right)}+c_{i}\right),\\ -\frac{\partial \log P\left(v\right)}{\partial c_{i}}=E_{v}\left[P\left(h_{i}\;|\;v\right)\right]-\text{sigm}\left(W_{i}\cdot v^{\left(i\right)}\right),\\ -\frac{\partial \log P\left(v\right)}{\partial b_{j}}=E_{v}\left[P\left(v_{j}\;|\;h\right)\right]-v_{j}^{\left(i\right)}. \end{array}$$

These gradients will be used in guiding the weight matrix's updates during the training procedure of the RBMs.

### 3.3. Sampling and Contrastive Divergence in an RBM

Using Gibbs sampling as the transition operator, samples of *p*(*x*) can be obtained by running a Markov chain to convergence [15]. To sample a joint of *n* random variables *X* = (*X*_1_,…, *X*_*n*_), Gibbs sampling performs a sequence of *n* sampling substeps of the form *X*_*i*_ ~ *P*(*X*_*i*_∣*X*_−*i*_), where *X*_−*i*_ represents the ensemble of the *n* − 1 random variables in *X* other than *X*_*i*_.

**Figure 2:**
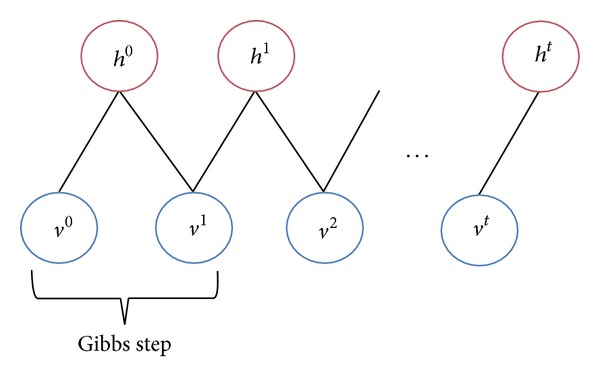
Gibbs sampling.

An RBM consists of visible and hidden units. However, since they are conditionally independent, we can perform block Gibbs sampling [15]. In this condition, hidden units are sampled simultaneously when given fixed values of the visible units. Similarly, visible units are sampled simultaneously when given the hidden units. A step in the Markov chain is thus taken as follows [15]:

(5) $$\begin{array}{c} h^{\left(n\right)}\sim \text{sigm}\left(W^{\prime }\cdot v^{\left(n\right)}+c\right),\\ v^{\left(n+1\right)}\sim \text{sigm}\left(W\cdot h^{\left(n\right)}+b\right), \end{array}$$

where *h* ^(*n*)^ refers to the set of all hidden units at the *n*th step of the Markov chain. What it means is that, for example, *h*_*i*_^(*n*)^ is randomly chosen to be 1 (versus 0) with probability sigm(*W*_*i*_′*v* ^(*n*)^ + *c*_*i*_), and similarly *v*_*j*_^(*n*+1)^ is randomly chosen to be 1 (versus 0) with probability sigm(*W*_*j*_ *h* ^(*n*)^ + *b*_*j*_) [15]. This can be illustrated graphically in Figure 2. Contrastive divergence does not wait for the chain to converge. Samples are obtained only after* k*-steps of Gibbs sampling. In practice, *k* = 1 has been shown to work surprisingly well [15].

### 3.4. ElGamal Scheme

In our privacy-preserving scheme, we use ElGamal [19], which is a typical public encryption method, as our cryptograph tool. Reference [20] has shown that the ElGamal encryption scheme is semantically secure [21] under a standard cryptographic assumption. In [12], the authors develop an elegant secure computing sigmoid function method and a secure computing product of two integer algorithms based on ElGamal's homomorphic property and probabilistic property. Here we give a brief review of these two algorithms. As shown in Algorithm 1, first Party *A* computes that *y*(*x*_1_ + *i*) − *R*, and *i* is all the possible input of Party *B*. Specifically, *y* is the sigmoid function. Similarly, as shown in Algorithm 2, Party *A* holds *M* and Party *B* holds *N*. Party *A* computes *M* × *i* for all possible inputs of Party *B* and then sends all encrypted messages to Party *B*. Then, Party *A* and Party *B* can obtain the secret share of *M* × *N* [12].

## 4. Privacy-Preserving Restricted Boltzmann Machine

### 4.1. Overview and Algorithm of Our Privacy-Preserving Restricted Boltzmann Machine

In order to use cryptographic tools in our privacy-preserving RBM, we use probability as the value of the hidden unit and visible unit. That means that when we are undertaking the Gibbs sampling process, we use the probability instead of {0,1} as the value of the hidden unit and visible unit. Therefore, we can use the ElGamal scheme to encrypt the probability after rounding the decimal. However, there will be some accuracy loss when we use this approximation. We will evaluate this accuracy loss in Section 5.

In our privacy-preserving RBM training algorithm, we assume the data are vertically partitioned. That means that each party owns some features of the dataset. Our privacy-preserving RBM is the first work on training restricted Boltzmann machine over a vertically partitioned dataset. We will look in detail at our training algorithm.

For each training iteration, two parties, *A* and *B*, own the inputs *v*_*A*_^0^ = (*v*_1_^0^, *v*_2_^0^,…, *v*_*m*_*A*__^0^) and *v*_*B*_^0^ = *v*_*m*_*A*_+1_,…, *v*_*m*_*A*_+*m*_*B*__ separately. The main idea of our privacy-preserving RBM is that when training our model, we use the cryptograph method (Algorithms 1 and 2) [12] to secure each step without revealing the original data to each other's party.

First, we let each party sum up their visible data of each sample. Then Party *A* computes sigmoid(∑_*k*≤*m*_*A*__(*w*_*jk*_ *v*_*k*_^0^ + *c*_*k*_) + *i*) − *R* for all possible *i*, where *R* is a random number generated by Party *A*. Then Party *A* rounds all these results to the integer and encrypts them. Then Party *A* sends the cipher message to Party *B* in the increasing order of *i*. Then Party *B* picks *i*, which is their sum-up value, rerandomizes it, and sends it to Party *A*, who partially decrypts this message and sends it back to Party *B*, who decrypts it and gets the value of sigmoid(∑_*k*≤*m*_*A*__(*w*_*jk*_ *v*_*k*_^0^ + *c*_*k*_) + ∑_*m*_*A*_≤*k*≤*m*_*A*_+*m*_*B*__(*w*_*jk*_ *v*_*k*_^0^ + *c*_*k*_)) − *R*. Specifically, *h*_*j*1_^0^ is *R* and *h*_*j*2_^0^ = sigmoid(∑_*k*≤*m*_*A*__(*w*_*jk*_ *v*_*k*_^0^ + *c*_*k*_) + ∑_*m*_*A*_≤*k*≤*m*_*A*_+*m*_*B*__(*w*_*jk*_ *v*_*k*_^0^ + *c*_*k*_)) − *R* as shown in the Privacy-Preserving Distributed Algorithm for RBM. Then, using the same method we can perform the rest of the privacy-preserving Gibbs sampling process.

For the second updating weight part, we use Algorithm 2 [12] to securely compute the products *v* ^0^ *h* ^0^ and *v* ^1^ *h* ^1^ separately. Specifically, *h* ^0^ = *h*_1_^0^ + *h*_2_^0^, *v* ^1^ = *v*_1_^1^ + *v*_2_^1^, and *h* ^1^ = *h*_1_^1^ + *h*_2_^1^, where the number on the top indicates the Gibbs step and the number on the bottom indicates the party the data belongs to. So we can get *v* ^0^ *h* ^0^ − *v* ^1^ *h* ^1^ = *v* ^0^(*h*_1_^0^ + *h*_2_^0^)−(*v*_1_^1^ + *v*_2_^1^)(*h*_1_^1^ + *h*_2_^1^). Regardless of which party *v* ^0^ belongs to, we can get the same result. Furthermore, we get *v* ^0^ *h* ^0^ − *v* ^1^ *h* ^1^ = *v* ^0^ *h*_1_^0^ + *v* ^0^ *h*_2_^0^ − *v*_1_^1^ *h*_1_^1^ − *v*_1_^1^ *h*_2_^1^ − *v*_2_^1^ *h*_1_^1^ − *v*_2_^1^ *h*_2_^1^. Therefore, we use Algorithm 2 to securely compute these products. As one example, *v*_1_^0^ *h*_2_^0^ indicates that *v*_1_^0^ belongs to Party *A*, which computes all *v*_1_^0^ × *i* − *R*′ for all *i*, rounds all these result to the integer and encrypts them, and then sends the cipher message to Party *B* in the increasing order of *i*. Then Party *B* picks *i*, which is their *h*_2_^0^ value, rerandomizes it, and sends it to Party *A*, who partially decrypts this message and sends it back to Party *B*, who decrypts it and gets the value of *v*_1_^0^ *h*_2_^0^ − *R*′. Specifically, *r*_11_^0^ is *R* and *r*_12_^0^ = *v*_1_^0^ *h*_2_^0^ − *R*′ as shown in the Privacy-Preserving Distributed Algorithm for RBM (Algorithm 3). Then, using the same method, we can perform the rest of the privacy-preserving product process.

Lastly, if Party *A* owns *v* ^0^, it can compute *v*_1_^0^ *h*_1_^0^ + *r*_11_^0^ − *v*_1_^1^ *h*_1_^1^ − *r*_11_^1^ − *r*_21_^1^, and Party *B* computes *r*_12_^0^ − *v*_2_^1^ *h*_2_^1^ − *r*_12_^1^ − *r*_22_^1^. Then Party *B* sends this to Party *A*, and Party *A* sums up these two to get the final value of *v* ^0^ *h* ^0^ − *v* ^1^ *h* ^1^. Then Party *A* can perform gradient descent to update the weight. Using the same method, we can update the bias of visible unit *b* and the bias of hidden unit *c*.

A privacy-preserving testing algorithm can be easily derived from the Gibbs sampling part of the privacy-preserving training algorithm.

### 4.2. Analysis of Algorithm Complexity and Accuracy Loss

The running time of one iteration of training consists of two parts, the Gibbs sampling and updating the weights. First, we analyze the execution time of the Gibbs sampling process. According to [12], Algorithm 1 takes *T* = (2 × Domain + 1)*E* + 2*D*, where Domain is the total number of *i* in Algorithm 1 and* E* and* D* are the costs of encryption and decryption. Therefore, in the Gibbs sampling process, we assume there are *S* samples, *H* hidden units, and *V* visible units. We can get the time cost as *H* × *T* + *V* × *T* + *H* × *T* = (2*H* + *V*)[(2 × Domain + 1)*E* + 2*D*].

In the updating weights process, Algorithm 2 also takes *T* = (2 × Domain + 1)*E* + 2*D*. Therefore, the total time used to encrypt and decrypt is 2 × *H* × *V* × *T* = 2*HV*[(2 × Domain + 1)*E* + 2*D*].

Combining the time for the two stages, we obtain the running time of one round of privacy-preserving RBM learning as (2*H* + *V* + 2*HV*)*T* = (2*H* + *V* + 2*HV*)  [(2 × Domain + 1)*E* + 2*D*].

In order to provide the preservation of privacy, we introduced two approximations in our algorithm. First, we replaced the binary value by the probability. Second, we mapped the real numbers to fixed-point representations to enable the cryptographic operations in Algorithms 1 and 2 [12]. This is necessary in that intermediate results, such as the values of visible and hidden units, are represented as real numbers in normal RBM learning, but cryptographic operations are on discrete finite fields. We will empirically evaluate the impact of these two sources of approximation on the accuracy loss of our RBM learning algorithm in Section 6. Below we give a brief theoretical analysis of the accuracy loss caused by the fixed-point representations. We assume that the error ratio bound which is caused by truncating the real number is *ϵ*. In the Gibbs sampling process, Algorithm 1 is applied three times; therefore, the error ratio bound is (1 + *ϵ*)^3^ − 1. In updating the weight process, Algorithm 2 is one for each dataset. The error ratio bound for *W* is *ϵ*.

### 4.3. Analysis of Algorithm's Security

In our distributed RBM training algorithm, except the computations that can be done by a party itself, all other computations that have to be done jointly by the two parties protect their input data with semantically secure encryptions. In addition, all intermediate computing results are also protected using the secret sharing scheme. In the semihonest model, both parties follow the algorithm without any deviation; our algorithm guarantees that the additional knowledge gained from the execution of our algorithm by a party is only the final training result. Therefore, our algorithm protects both parties' privacy in this model.

## 5. Experiments

In this section, we explain the experimental process for measuring the accuracy loss of our modified algorithms. We compare the testing error rates to non-privacy-preserving cases. In the second set, we distinguish two types of approximations introduced by our algorithms: a conversion of real numbers to fixed-point numbers when applying cryptographic algorithms and an analysis of how they affect the accuracy of the RBM.

### 5.1. Setup

The algorithms were implemented in MATLAB. The experiments were executed on a Windows computer with a core i5 2.3 GHz Intel processor and 3 Gb of memory. The testing datasets were MINST database of handwritten digits. We chose the number of hidden nodes based on the number of attributes. Weights were initialized as uniformly random values in the range of [−0.1, 0.1]. Feature values in each dataset were normalized between 0 and 1.

### 5.2. Effects of Two Types of Approximation on Accuracy

In this section, we evaluate the loss of accuracy of our modified training model. In our model, there exist two approximations. The first one is that we use probability instead of binary value as our Gibbs sampling result. The second is that we truncate the probability to finite digits so that we can shift the decimal point and then use this number for encryption. We then distinguish and evaluate the effects of these two approximation types without cryptographic operations (we call it approximation test).

**Figure 3:**
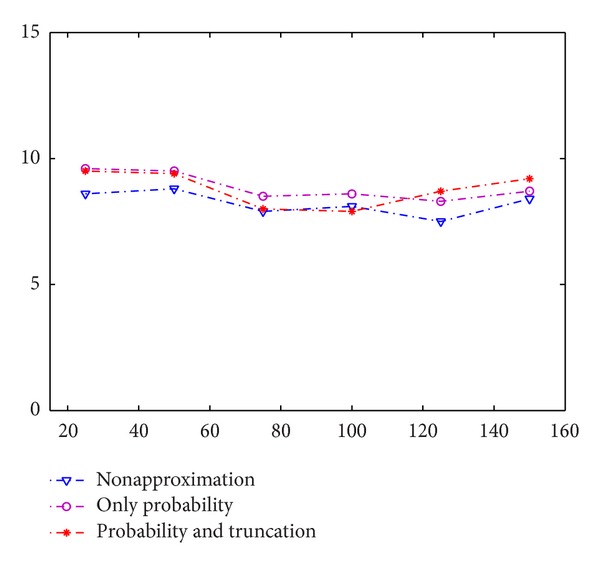
The error rates on training epochs.

First, we compare the loss of accuracy caused by using probability instead of binary value on the MNIST dataset. We chose 5,000 samples as training data and 1,000 as testing data. We then set the 100 hidden units and perform the experiments by varying the number of epochs and evaluating the loss of accuracy on different training epochs. In Figure 3, we can see that the accuracy caused by this approximation is less than 1%. Since encryption and decryption do not influence the accuracy of our model, this is the accurate amount of loss of our privacy-preserving training method.

Second, we compare the accuracy loss caused by truncating the probability to finite digits. Specifically, we truncate the number to two digits. We set the parameter as the same as the first experiment. From the results we can see that the error rate is still close to the algorithm without approximation.

## 6. Conclusion and Future Work

In this paper, we have presented a privacy-preserving algorithm for RBM. The algorithm guarantees privacy in a standard cryptographic model, the semihonest model. Although approximations are introduced in the algorithm, the experiments on real-world data show that the amount of accuracy loss is reasonable.

Using our techniques, it should not be difficult to develop the privacy-preserving algorithms for RBM learning with three or more participants. In this paper, we have proposed only the RBM training method. A future research topic would be to apply it in a practical implementation and to extend our work to deep networks training.

## Figures and Tables

**Algorithm 1:**
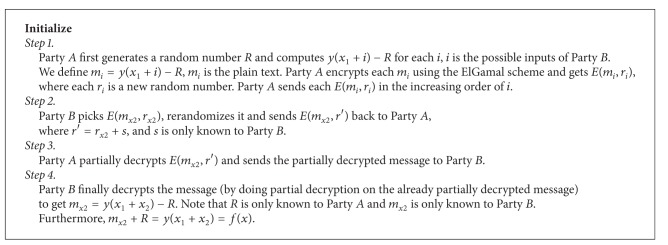
Securely computing the sigmoid function [12].

**Algorithm 2:**
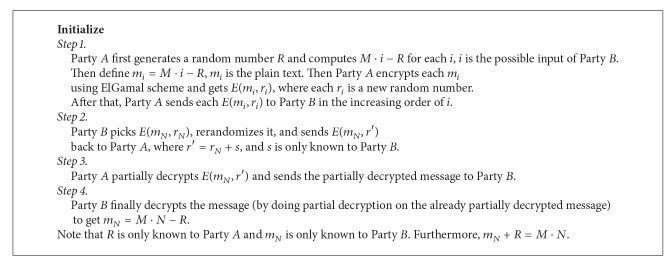
Securely computing the product of two integers [12].

**Algorithm 3:**
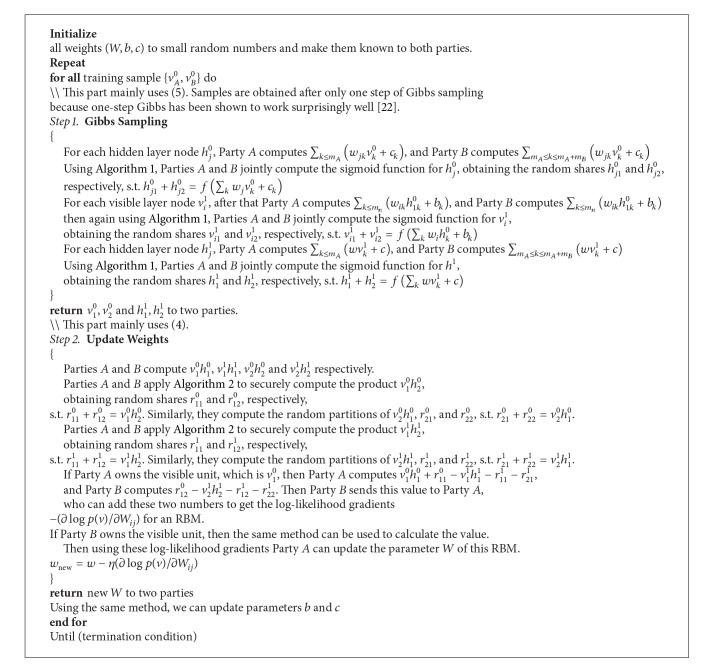
Privacy-Preserving Distributed Algorithm for RBM.
